# Beyond the 72 h window: operationalizing safe abortion as essential care for sexual violence survivors in humanitarian settings

**DOI:** 10.3389/frph.2026.1809707

**Published:** 2026-05-13

**Authors:** Simon Binezero Mambo, Baudouine Akonkwa Mweze, Ibrahim Lubaliro, Moise Byamungu, Nadia Jive Lobo, Beniel Ulrich Agossou, Gloria Neema Bizimana

**Affiliations:** 1 Youth Alliance for Reproductive Health, Goma, Democratic Republic of Congo; 2 Departement of Public Health, School of Allied Health Sciences, Kampala International University Western-Campus, Kampala, Uganda; 3 Sexual and Reproductive Health Working Group, Goma, Democratic Republic of Congo; 4 Ipas RDC, Kinshasa, Democratic Republic of Congo; 5 Centre ODAS, Abidjan, Côte D'Ivoire; 6 Department of Pediatric and Child Health, Faculty of Clinical Medicine and Dentistry, Kampala International University Western-Campus, Kampala, Uganda

**Keywords:** beyond 72 h, humanitarian settings, post-rape care, safe abortion, sexual violence

## Abstract

In the Democratic Republic of Congo (DRC), sexual violence is systematically employed as a weapon of war, precipitating a profound reproductive health crisis. Current clinical protocols prioritize intervention within a 72-hour window; however, pervasive insecurity, displacement, and geographic barriers frequently prevent survivors from accessing care within this timeframe. This broken link in the referral chain effectively denies survivors safe abortion services, resulting in forced pregnancies and secondary institutional trauma. This review evaluates the DRC's humanitarian response by analyzing the integration of the Minimum Initial Service Package (MISP) against evolving domestic legislation, specifically the 2018 gazetting of the Maputo Protocol and the 2020 National Comprehensive Abortion Care Guidelines. Despite a supreme legal mandate under Article 14 of the Maputo Protocol authorizing safe abortion for survivors, implementation is obstructed by provider stigma, the misuse of conscientious objection, and a hierarchy of services that prioritizes physical trauma repair over reproductive autonomy. Clinical pathways for cases presenting beyond the 72-hour emergency window remain poorly operationalized. To bridge the gap between legal rights and clinical reality, the DRC must transition toward a Comprehensive Clinical Management model. This requires institutionalizing task-sharing with midwives and integrating the Addressing Reproductive Coercion in Health Settings (ARCHES) framework to manage reproductive violence beyond the emergency phase. Safe abortion must be repositioned as an essential, non-optional component of post-rape care. True recovery depends on aligning clinical practice with international human rights standards, ensuring reproductive self-determination is upheld amidst protracted crisis and systemic instability.

## Navigating legal mandates and humanitarian crises for sexual violence survivors in the DRC

Across the globe, sexual violence is systematically employed by state armies, non-state armed groups, and terrorist organizations as a deliberate tactic of war (1, 2). It is used to humiliate, dominate, and displace communities, often targeting specific ethnic groups to sow terror and control reproduction. In the Democratic Republic of Congo (DRC), rape has been one of the most prolific weapons of conflict since 1998, reaching an unparalleled scale. Women and girls navigate this violence within a societal framework where their status is traditionally and legally marginalized, often leading to a culture of silence and systemic impunity, even when complaints are filed, prosecutions remain rare due to outdated laws and a refusal to recognize the gravity of sexual offenses (1, 3).

For nearly three decades, the eastern provinces of North Kivu, South Kivu, and Ituri have been scarred by a cycle of armed conflict (1, 3). This environment has catalyzed a “metamorphosis in criminality,” where the female body has become a literal battlefield for ethnic and political tensions. The human cost has reached staggering levels: over 120 armed groups operate with near-total impunity, and displaced survivors in informal camps face extreme risks. Data from Médecins Sans Frontières (MSF) reveals a harrowing reality: 84% of sexual violence victims in certain regions were assaulted while performing daily survival tasks, such as fetching water or firewood (1). In 2023 alone, MSF treated an unprecedented 25,166 survivors in DRC; among them, 8,115 women sought abortion services, with 94% of these requests coming from displacement sites around Goma (2). This systematic use of sexual violence as a weapon of war inflicts profound physical and psychological trauma, high rates of STI transmission, and unwanted pregnancies, particularly among displaced populations.

The clinical care offered to survivors is theoretically guided by WHO and Inter-Agency Reproductive Health (IARH) standards. While IARH Kit 3 provides Post-Exposure Prophylaxis (PEP) and Emergency Contraception (EC), these essential interventions are strictly time-bound. For survivors, accessing emergency contraception is most effective within 72 h and loses significant efficacy after 120 h (3). Unfortunately, the humanitarian reality in the DRC often prevents survivors from reaching clinics within this 5-day window due to insecurity and long distances (4–6). When a survivor presents after 120 h, the clinical focus must shift from prevention to the management of potential pregnancy. Failure to provide safe alternatives often leads to ostracization, infanticide, or dangerous clandestine abortions.

Despite clear medical needs, a profound legal-clinical schism persists in the DRC. While the 2018 gazetting of the Maputo Protocol provides a supreme legal mandate for safe abortion in cases of rape, local interpretations remain restrictive (6–8). Many providers, anchored in outdated penal codes, hesitate to offer safe abortion care due to a lack of training or fear of legal repercussions. This hesitation often stems from a misunderstanding of the DRC's Monist legal system; under this system, once an international treaty like the Maputo Protocol is ratified and gazetted, it automatically becomes national law with a higher authority than the domestic Penal Code (7, 8). In the DRC's hierarchy of norms, the Maputo Protocol sits above the 1940 Penal Code, meaning its provisions legally override any conflicting or restrictive local statutes. Therefore, the Protocol does not need to be physically written into the Penal Code to be the law of the land. For clinicians, this means that providing safe abortion care to survivors is not a violation of the law, but rather the fulfillment of a superior legal mandate that protects both the patient's rights and the provider's clinical practice (7–9). This creates a silent exclusion,” where the most vulnerable are denied the care the law is supposed to guarantee.

The historical evolution of Congolese law shows progress, but implementation lags. The 2006 Sexual Violence Law expanded the definition of rape to include various forms of penetration and coercive contexts, and the 2020 National Standards and Guidelines for Comprehensive Abortion Care (SCACF) explicitly state that victims of rape must be treated with specialized care (4). This review argues that safe abortion must be integrated as an essential, non-optional component of the “clinical management of rape” (CMR) in humanitarian settings (8, 10, 11). By synchronizing clinical practice with the Maputo Protocol, the DRC can move toward a model of care that respects survivor autonomy and addresses the long-term biological and psychological consequences of sexual violence. Restoring reproductive self-determination is both a moral and legal imperative that cannot be ignored, even amidst the complexities of a protracted crisis.

## The implementation gap: deciphering the “broken link” between legal mandates and service delivery

Despite clear medical needs, a profound legal-clinical schism persists in the DRC. A comprehensive response for survivors of Gender-Based Violence (GBV) requires the integration of Psychosocial Support and Case Management. This approach provides emotional stabilization and coordinated access to multisectoral needs through safe referral pathways tailored to age, gender, and disability. From a clinical perspective, medical services must prioritize urgent care, including the management of injuries and the administration of Post-Exposure Prophylaxis (PEP) and emergency contraception within critical timeframes (7, 10, 11). Simultaneously, specialized mental health services are essential to treat the deep-seated trauma and psychological disorders resulting from assault. To ensure long-term recovery, legal and judicial assistance must empower survivors to claim their rights, while safe shelters and security services provide the necessary physical protection and confidentiality. Socio-economic empowerment initiatives build resilience through skills training, reducing dependency and the risk of further exploitation while restoring the survivor's dignity (10).

Despite this robust normative framework, a significant implementation gap persists. Deep-seated provider stigma, the misuse of conscientious objection, and a pervasive lack of survivor awareness regarding their rights create secondary barriers to care (5). This structural failure often results in the “silent exclusion” of safe abortion and comprehensive reproductive health from emergency medical responses, even when such services are legally mandated.

The evolution of Congolese legislation regarding sexual violence is marked by two fundamental shifts. Initially, the legal definition of rape was highly restrictive, limited solely to vaginal penetration. However, this changed with the enactment of Law No. 06/018 of July 20, 2006, which amended Article 170 of the Penal Code to significantly broaden this scope.

Under this 2006 reform, the legal definition of rape was expanded beyond traditional penetration to include any act of sexual penetration whether vaginal, anal, or oral committed by the use of force, threats, or coercion, as well as the use of objects. This legislative shift was a critical response to the metamorphosis of criminality documented during the country's protracted conflicts, ensuring that the law recognized the diverse and brutal realities of sexual violence faced by survivors. The law now recognizes that any sexual act including superficial penetration which is a critical legal and clinical distinction designed to protect survivors who may not have experienced full intercourse but were still subjected to a violation of their bodily integrity, oral or anal intromission, or the brutal use of objects constitutes rape. This revision was essential to address the horrific metamorphosis of criminality documented during decades of armed conflict. Furthermore, the law is now gender-neutral, acknowledging that both men and women can be victims or perpetrators, thereby expanding the protective umbrella of the judicial system (6, 12). Unlike the restrictive 1940 Penal Code, the Maputo Protocol serves as the supreme legal instrument for women's rights in the country. By publishing the Protocol in its Official Journal, the DRC effectively integrated Article 14 into its domestic legal order. This article is revolutionary: it explicitly mandates the authorization of safe abortion in cases of sexual assault, rape, incest, and when the pregnancy endangers the mental or physical health of the mother or the life of the fetus (8).

The Protocol creates a non-negotiable link between survivor care and abortion access, recognizing that the denial of safe abortion services constitutes a form of continued institutional violence. In the DRC, this means that the clinical management of rape must, by law, include the option of safe abortion. However, a legal-clinical schism remains; many healthcare providers stay anchored in outdated penal codes, unaware that the Maputo Protocol takes precedence over contrary domestic laws (8, 9).

To operationalize these definitions, the DRC has adopted additional measures, including the 2006 Sexual Violence Laws and the 2015 integration of the Rome Statute. A pivotal advancement occurred in December 2022 with the promulgation of the Reparations Law, the National Fund for Reparations for Victims (FONAREV). Its mission is to identify, support, and provide reparations to victims of conflict-related sexual violence, as well as other serious crimes against peace and the security of mankind (6, 12). Yet, without the systematic integration of reproductive health services specifically safe abortion these reparations remain incomplete, addressing judicial harm while neglecting the biological and psychological consequences of the assault.

The status quo in the DRC is a stark reminder of the consequences of institutional neglect. To bridge this gap, the Maputo Protocol must transition from a document in the Official Journal to a lived reality in every health center. True accountability and healing for survivors will only be achieved when societal stigma is deconstructed and legal rights are mirrored by accessible, high-quality clinical services. Restoring reproductive self-determination is both a moral and legal imperative that cannot be ignored, even amidst the complexities of a protracted crisis Table 1: Shows the Chronological Legal Framework: Status, Reparations, and Access.

**Table 1:** Chronological legal framework: status, reparations, and access.

Year	Legal instrument	Status & authority level	Impact on SGBV & survivor access
1940	Penal code (decree of Jan 30)	Subordinate/colonial era: remains on the books but is superseded by the constitution and international treaties.	Criminalized abortion in all cases. It remains the source of “provider fear” and institutional stigma.
2006	Sexual violence law (Law no. 06/018)	Active national law: primary legislation for prosecuting sexual crimes.	Broadened the definition of rape and removed the “penetration only” requirement. Recognized sexual slavery.
2015	Penal code amendment (Law no. 15/021)	Active national law: harmonized domestic law with the Rome statute.	Formally classified SGBV as a war crime or crime against humanity in conflict settings, making these crimes imprescriptible.
2018	Maputo protocol (gazetted March 14)	Supreme legal mandate: under the DRC's monist system, it takes precedence over the 1940 penal code.	Legalized safe abortion (Art. 14) for rape, incest, and health risks. It is the ultimate “shield” for clinical providers.
2020	SCACF guidelines	Administrative mandate: issued by the ministry of health to operationalize the Maputo protocol.	Standardized self-attestation as the only requirement for care. Removed the need for police reports or judicial proof.
2022	Reparations law (Law no. 22/065)	Special national law: established the legal framework for the FONAREV fund.	Grants victims of conflict-related SGBV the right to reparation and protection without requiring a prior criminal conviction.

## Bridging the gap: evaluating the integration of safe abortion care within the MISP framework in humanitarian settings

The successive armed conflicts that have scarred the history of the Democratic Republic of Congo (DRC) have resulted in more than five million lives lost (8). Beyond the staggering death toll, hundreds of thousands of women and girls have fallen victim to systemic sexual violence. The forced recruitment of children and the intermingling of various armed groups have served to spread these atrocities across the national territory, often used as a tool for pillaging resources or destabilizing communities (1, 3). This violence is further compounded by retrograde customs, discriminatory legal texts, and sexist social practices that entrench the inferior status of women and minors in both conflict and domestic settings.

In humanitarian crises, mass population displacement and the collapse of social protection systems leave refugees and Internally Displaced Persons (IDPs) exceptionally vulnerable. Sexual violence in these contexts constitutes a grave violation of International Humanitarian Law and human rights (1, 6). Under the Minimum Initial Service Package (MISP) for sexual and reproductive health, the medical management of survivors is categorized as a life-saving emergency. The MISP dictates that care must be holistic, rapid, and compassionate (9). However, in practice, a “hierarchy of services” often emerges where immediate surgical repairs or pain management are prioritized, while the critical 72-hour window for Post-Exposure Prophylaxis (PEP), STI prophylaxis, and Emergency Contraception (EC) remains inconsistently implemented across volatile zones (9, 13).

Central to the clinical response is the right to safe abortion for survivors whose pregnancies result from rape. This is not an elective procedure; it is an essential component of reproductive autonomy and mental health protection, as recognized by the Maputo Protocol (5). To be effective, this care must be integrated into a One-Stop clinical pathway that includes vaccinations, forensic evidence collection, and psychological support. In the volatile environment, it is vital to decentralize these services, ensuring that survivors in remote displacement camps can access safe abortion without facing the risks of traveling to urban centers (14).

Despite established protocols, delivery is hampered by structural deficiencies within the Congolese health system, including chronic shortages of “Rape Kits” and medical abortion commodities. Furthermore, many NGO-led responses practice a “silent exclusion” of safe abortion services. This is often driven by providers who exercise conscientious objection or prioritize STI prevention due to personal biases (5). Consequently, the quality of care is frequently dictated by an individual provider's attitude rather than standardized medical ethics, leaving survivors at risk of forced pregnancies and further trauma.

Beyond the clinical setting, the response is weakened by fragmented coordination and deep-seated community barriers. Survivors often reach health centers long after the 72- or 120-hour windows have closed, paralyzed by the fear of social and religious stigmatization. Many humanitarian projects focus heavily on psychosocial recovery while neglecting the immediate clinical requirement of safe abortion. This fragmentation prevents the “Holistic Care Path”—pioneered by models like Panzi Hospital from being successfully replicated in frontline humanitarian corridors, leaving a significant gap in the recovery and dignity of women and girls in the DRC (11).

**Figure 1:**
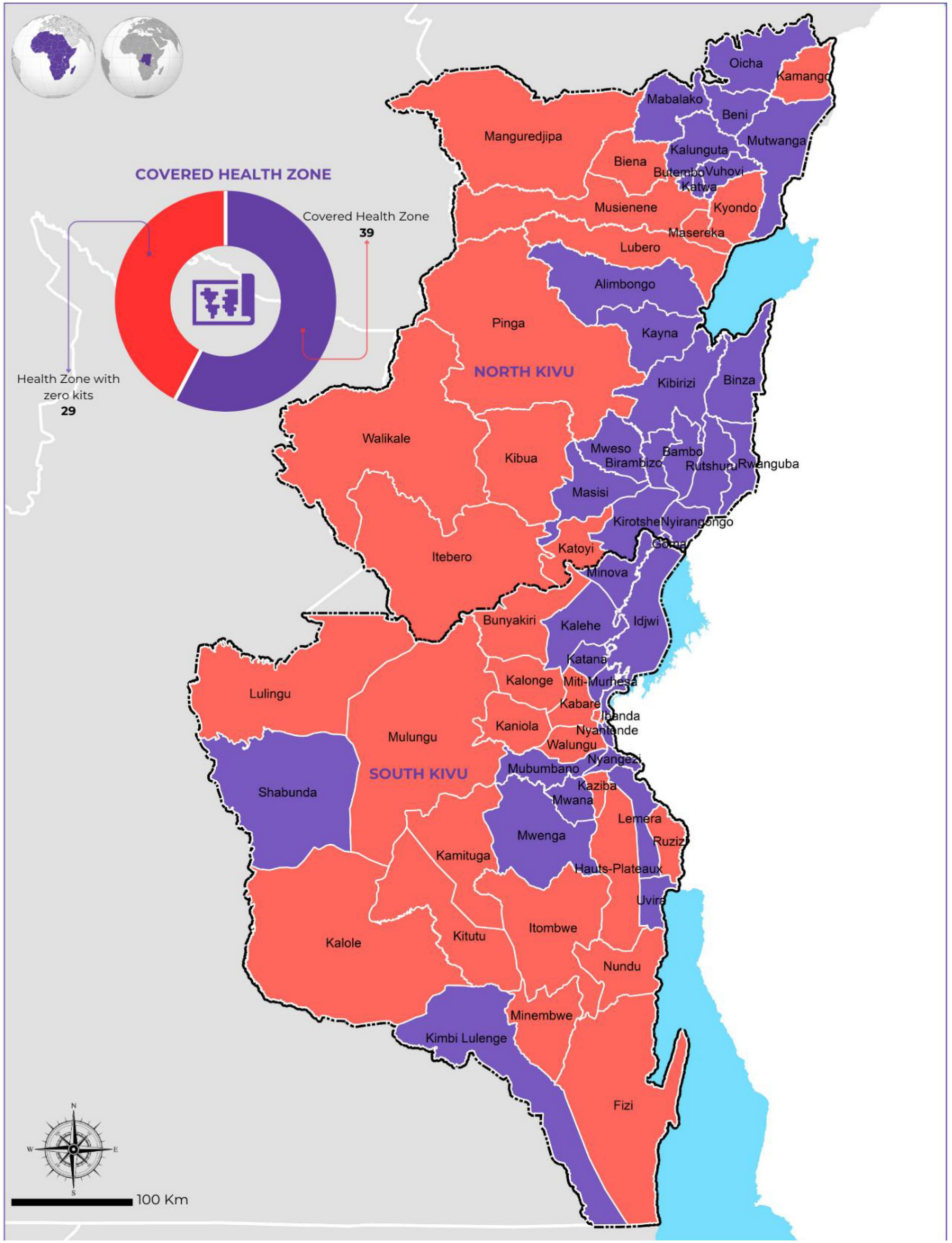
MAP of health zone with post-rape services in north and south Kivu.

To safeguard the safety and dignity of survivors, Congolese law (including Law No. 22/065 of December 2022) mandates that judicial officers and judges handle sexual violence cases with absolute discretion, including the use of in camera proceedings (12, 15). Throughout the legal process, survivors are entitled to the assistance of counsel and a psychologist. However, these legal protections remain incomplete without the parallel provision of reproductive health services, as a survivor's recovery is inextricably linked to their ability to access the full range of medical care mandated by international and national standards. Despite the legal and judicial safeguards, the operational reality on the ground as illustrated in Figure 1 reveals significant gaps in the availability of post-rape services across North and South Kivu. While specific prevalence data of sexual violence per health zone is currently unavailable, the lack of essential kits in many areas is largely due to the absence of government administration and the limited presence of partners in regions where security challenges restrict humanitarian access.

## Strategic integration: pathways to comprehensive clinical management and task-sharing for safe abortion

To address the critical health needs of survivors in the Democratic Republic of the Congo (DRC), a transformative clinical model is required to transition safe abortion from a siloed service into an integrated component of the post-rape clinical pathway. Central to this shift is the strategic adoption of the ARCHES framework (Addressing Reproductive Coercion in Health Settings), which provides a survivor-centered model to identify and respond to reproductive violence, ensuring that clinical interventions are both protective and empowering (12).

This approach necessitates two non-negotiable pillars of care: meeting the survivor's comprehensive health needs—including injury treatment, STI/HIV prophylaxis, and psychological stabilization while simultaneously ensuring the meticulous collection of forensic evidence to support the pursuit of justice.

The effectiveness of this clinical response is heavily dictated by time. According to WHO standards, survivors must be offered Emergency Contraception (EC) as soon as possible, ideally within 72 h, though it remains a viable option for up to 120 h (7, 10). However, when a survivor presents after this 5-day window, the clinical focus must shift from prevention to the management of potential pregnancy. In a nation where forced pregnancy constitutes a secondary trauma, immediate access to emotional support and clear information regarding safe abortion is vital. This is particularly urgent in the DRC, where nearly 16,000 women die annually from pregnancy-related complications—a crisis exacerbated by the metamorphosis of criminality in conflict zones (5).

Despite this medical urgency, a profound legal-clinical schism persists. While the 2018 gazetting of the Maputo Protocol provides a supreme legal mandate for safe abortion in cases of rape, local interpretations remain restrictive. Many providers, anchored in outdated penal codes, hesitate to offer care due to a lack of training or fear of legal repercussions (8, 9). This hesitation often stems from a misunderstanding of the DRC's Monist legal system. In the DRC's hierarchy of norms, the Maputo Protocol automatically becomes national law upon gazetting and holds higher authority than the domestic Penal Code (8, 9). It does not need to be physically written into the Penal Code to be enforceable. For clinicians, providing safe abortion care is not a violation of the law, but rather the fulfillment of a superior legal mandate (14). To bridge the gap between medical necessity and the law, the DRC must operationalize task-sharing. By adopting WHO guidelines, the DRC authorizes mid-level providersspecifically midwives, nurses, and medical assistants to provide safe abortion care across all levels of the healthcare pyramid (10, 11).

Empowering service providers requires more than policy change; it requires competency-based training in Medical Abortion (MA) and Manual Vacuum Aspiration (MVA) (8, 14). Mid-level providers including midwives, nurses, and medical assistants who are trained to perform clinical tasks traditionally reserved for physicians are often the only point of contact in displacement camps and rural health posts. True empowerment for these clinicians necessitates a dual approach: technical mastery of life-saving procedures and participation in Value Clarification and Transformation (VCAT) sessions. These transformative sessions are essential to address provider stigma, allowing clinicians to deconstruct personal biases and navigate the tension between social norms and professional ethics. By shifting the focus from judgment to empathy and legal duty, VCAT ensures that trained providers are not only technically capable but also morally supported to deliver safe abortion care without obstruction.

Decentralizing authority to these frontline workers ensures that care is available at the point of need, bypassing chronic physician shortages (12, 14). Access to care is impossible without a guaranteed supply of commodities. Last-mile supply chainsthe final and most critical stage of the logistics network where medical commodities are moved from regional hubs to the frontline health centers where survivors receive care must be secured to prevent stockouts of Inter-Agency Reproductive Health (IARH) Kits (2, 4). This requires integrating MA medications (Mifepristone and Misoprostol) into the standard post-rape care kits distributed to primary health centers (13).

Operationalizing this requires a push pull logistics system, where community-based midwives can request refills based on real-time consumption data without urban administrative hurdles. Moving from theoretical access to actual service delivery depends entirely on the physical presence of these commodities in remote conflict zones (16, 17).

Addressing provider stigma and the misuse of conscientious objection is the most significant operational hurdle. This can be achieved through Values Clarification and Attitude Transformation (VCAT) workshops (8). These sessions allow providers to separate personal beliefs from professional and legal obligations. Furthermore, integrating the ARCHES framework allows providers to identify ongoing reproductive coercion—such as partner interference with contraceptioneven after the initial assault (12). By using ARCHES, midwives can offer discreet contraceptive options and emotional support, ensuring that reproductive self-determination is maintained beyond the emergency phase. This moves the response beyond mere medical stabilization toward true, holistic healing and long-term recovery (16, 17). Table 2 is the Proposed Comprehensive Clinical Management model.

**Table 2:** Proposed comprehensive clinical management model.

Service category	Immediate response (0–72 h)	Comprehensive care (beyond 72 h)	Task-sharing & operational requirements
Pregnancy management	Prevention: emergency contraception (EC) or copper IUD.	Management: safe abortion (SCACF) as per Maputo protocol & 2020 guidelines.	Midwives/nurses: authorized to provide MA (mifepristone/misoprostol) and MVA.
HIV/STI protection	Prophylaxis: PEP (post-exposure prophylaxis) and STI presumptive treatment.	Screening: testing, long-term STI management, and ART if seroconversion occurs.	Health workers: decentralized testing and dispensing of PEP/STI kits at primary level.
Psychosocial support	Crisis intervention: psychological first aid and emotional stabilization.	Mental health care: long-term therapy for PTSD, depression, and stigma mitigation.	Social workers: trained in “survivor-centered” counseling and the ARCHES framework.
Legal & safety	Safety first: immediate referral to safe shelters and police protection.	Justice: ongoing legal assistance and support through the FONAREV process.	Paralegals: community-based referral to ensure “last-mile” access to judicial services.

## Conclusion

The clinical management of sexual violence survivors in the Democratic Republic of Congo (DRC) must undergo a fundamental paradigm shift, moving from a reactive, time-bound emergency intervention to a proactive, comprehensive care model that transcends the 72-hour window. The persistent “legal-clinical schism” leaves thousands of women and girls vulnerable to the secondary trauma of forced pregnancy, effectively nullifying the supreme legal protections afforded by the Maputo Protocol and the 2020 National SCACF Guidelines.

To bridge this implementation gap, the Ministry of Health and humanitarian stakeholders must formalize clinical protocols that recognize safe abortion not as an elective service, but as an essential, life-saving component of the holistic post-rape care pathway. By decentralizing these services particularly to displacement camps and frontline health posts the health system can finally address the long-term biological and psychological consequences of weaponized sexual violence that manifest when the prevention window is missed.

Operationalizing these mandates requires two urgent structural reforms: systematic task-sharing and the fortification of last-mile supply chains for medical abortion commodities. Empowering midwives and mid-level providers to deliver safe abortion care is the only viable strategy to circumvent the chronic shortage of specialized physicians in conflict-affected provinces. Furthermore, addressing deep-seated provider stigma and the misuse of conscientious objection through Value Clarification Action and Transformation is vital to ensure that a survivor's fundamental rights are not contingent upon an individual provider's personal bias. In a nation where the female body has long been utilized as a literal battlefield, restoring reproductive self-determination is both a moral and a legal imperative. Only by aligning clinical reality with international human rights standards can the DRC ensure the dignity, health, and long-term recovery of survivors navigating the complexities of a protracted crisis.
